# Three Small Molecule Entities (MPK18, MPK334 and YAK308) with Activity against *Haemonchus contortus* In Vitro

**DOI:** 10.3390/molecules26092819

**Published:** 2021-05-10

**Authors:** Aya C. Taki, Abdul Jabbar, Thomas Kurz, Beate Lungerich, Guangxu Ma, Joseph J. Byrne, Marc Pflieger, Yodita Asfaha, Fabian Fischer, Bill C. H. Chang, Brad E. Sleebs, Robin B. Gasser

**Affiliations:** 1 Department of Biosciences, Melbourne Veterinary School, Faculty of Veterinary and Agricultural Sciences, The University of Melbourne, Parkville 3010, Victoria, Australia; aya.taki@unimelb.edu.au (A.C.T.); jabbara@unimelb.edu.au (A.J.); guangxu.ma@unimelb.edu.au (G.M.); byrnej1@unimelb.edu.au (J.J.B.); bchang@yourgene.com.tw (B.C.H.C.); sleebs@wehi.edu.au (B.E.S.); 2 Institute of Pharmaceutical and Medicinal Chemistry, Heinrich-Heine-University Düsseldorf, 40225 Düsseldorf, Germany; thomas.kurz@hhu.de (T.K.); beate.lungerich@hhu.de (B.L.); pflieger@hhu.de (M.P.); yodita.asfaha@uni-duesseldorf.de (Y.A.); Fabian.Fischer.14@uni-duesseldorf.de (F.F.); 3 Walter and Eliza Hall Institute of Medical Research, Parkville 3052, Victoria, Australia; 4 Faculty of Medicine, Dentistry and Health Sciences, The University of Melbourne, Parkville 3010, Victoria, Australia

**Keywords:** *Haemonchus contortus*, phenotypic screening, anthelmintics, small molecules

## Abstract

Due to widespread multi-drug resistance in parasitic nematodes of livestock animals, there is an urgent need to discover new anthelmintics with distinct mechanisms of action. Extending previous work, here we screened a panel of 245 chemically-diverse small molecules for anti-parasitic activity against *Haemonchus contortus*—an economically important parasitic nematode of livestock. This panel was screened in vitro against exsheathed third-stage larvae (xL3) of *H. contortus* using an established phenotypic assay, and the potency of select compounds to inhibit larval motility and development assessed in dose-response assays. Of the 245 compounds screened, three—designated MPK18, MPK334 and YAK308—induced non-wildtype larval phenotypes and repeatedly inhibited xL3-motility, with IC_50_ values of 45.2 µM, 17.1 µM and 52.7 µM, respectively; two also inhibited larval development, with IC_50_ values of 12.3 µM (MPK334) and 6.5 µM (YAK308), and none of the three was toxic to human liver cells (HepG2). These findings suggest that these compounds deserve further evaluation as nematocidal candidates. Future work should focus on structure–activity relationship (SAR) studies of these chemical scaffolds, and assess the in vitro and in vivo efficacies and safety of optimised compounds against adults of *H. contortus*.

## 1. Introduction

Parasitic nematodes (order Strongylida) cause substantial mortality and morbidity in livestock animals worldwide, leading to major losses to the global food production annually. Gastrointestinal nematodes, such as species of *Haemonchus*, *Ostertagia*, *Teladorsagia*, *Trichostrongylus*, *Cooperia*, *Nematodirus* and *Oesophagostomum*, cause some of the most important diseases, affecting the health of hundreds of millions of animals (including sheep, goats, cattle and pigs), with very substantial economic losses every year [1,2,3]. These parasites cause gastroenteritis, anaemia and/or reduced feed conversion, weight gain and growth as well as weight loss, and death in severely affected animals [1].

The control of strongylid nematodes is mostly achieved through a suppressive or strategic use of anthelmintic drugs [4,5]. However, the excessive and relatively uncontrolled use of these drugs has led to resistance to one or more of the major compound classes, including benzimidazoles, imidazothiazoles, macrocyclic lactones, aminoacetonitrile derivatives, tetrahydropyrimidines, salicylanilides and organophosphates, and is now widespread [6]. As no vaccines are available to prevent most diseases caused by these worms, with the exception of haemonchosis caused by *Haemonchus contortus* (barber’s pole worm) (Barbervax; cf. [7]), there is a need to discover and develop new drugs to ensure effective and sustained control in the immediate future. Although, between 2008 and 2010, an aminoacetonitrile derivative (monepantel) and a spiroindole (derquantel) provided renewed hope for the development of new classes of synthetic or semi-synthetic nematocides [8,9,10], subsequent success in discovering compounds that kill a spectrum of key gastrointestinal nematode species of livestock has been very limited. Thus, there is continued need to discover new anthelmintic compounds with distinct mechanisms of action.

In the search for new anthelmintic candidates, our research group has developed industry-linked collaborations focused on the screening of synthetic and natural compound libraries [11,12,13] for activity and potency against *H. contortus*—a key representative of the Strongylida—in a semi-automated platform [14]. To date, this program has identified a panel of 35 compounds, including kinase inhibitors, tolfenpyrad (an approved pesticide), and α-pyrones, which have been critically assessed for their potential as anthelmintic candidates (reviewed by [12,13]). Here, we extend this work to evaluate a panel of a chemically diverse group of compounds (including tribenzylamine, quinoline, hydroxamic acid and hydrazonamide histone deacetylase inhibitors) which had been synthesised or designed with a focus on developing anti-infectives (against parasitic protists), anti-cancer (against glioblastoma or neuroblastoma) or blood pressure-reducing drug candidates (e.g., [15,16]). We evaluated the potency of active (“hit”) compounds against larvae of *H. contortus* to inhibit motility and development, as a basis for future structure-activity-relationship (SAR) studies.

## 2. Results and Discussion

### 2.1. Three Compounds Induced a Phenotypic Alteration in the Primary Screen

**Figure 1:**
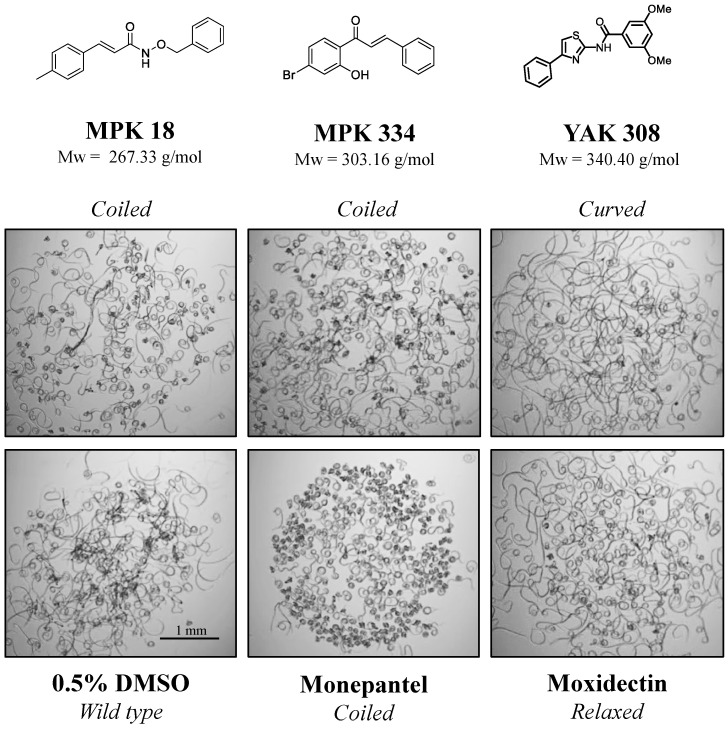
Three compounds identified to induce abnormal phenotypes in exsheathed third-stage larvae (xL3) of *Haemonchus contortus* in the primary screen. The representative single-frame images (25-times magnification) of short video (5 s) of xL3s captured in primary screen displaying non-wildtype phenotypes (“coiled” or “curved”) induced by each of the three hit compounds (MPK18, MPK334 and YAK304) at 20 µM after 72 h of exposure. The structures of three compounds are presented with their respective molecular mass (Mw). Two control compounds (monepantel and moxidectin) at 20 µM, and no compound control (0.5% DMSO), were used as references to the non-wildtype (“coiled” for monepantel) and wild-type phenotypes, respectively. MPK18- and MPK334-treated xL3s exhibited a “coiled” phenotype, similar to xL3s exposed to that of monepantel. YAK304-treated xL3 showed a “curved” phenotype, which was distinct from all controls.

The primary screening of 245 compounds identified three active “hits”. These compounds were 3-(p-tolyl)acrylamide (designated MPK18), a chalcone (MPK334) and 2-amino-4-phenylthiazole (YAK308), and all three compounds altered the phenotypes (morphology) of a proportion of xL3s after 72 h of exposure to each of compounds at 20 µM. A “coiled” phenotype was exhibited by ~50% of MPK18– and MPK334–treated xL3s based on a visual inspection of video recordings (Figure 1); this phenotype was similar to that of xL3s exposed to monepantel (positive control) over 72 h. The coiled xL3s were immotile and appeared to be dead; this deleterious effect was consistent with observations made for previously identified active compounds [17,18]. YAK308 induced a “curved” phenotype in ~90% of xL3s (Figure 1), distinct to phenotypes in all control wells (0.5% DMSO, monepantel and moxidectin). Although the curved phenotype seemed to be unique, a similar phenotype was observed previously in xL3s and L4s of *H. contortus* exposed in vitro to compound HBK4—an active benzimidazole-derivative from a previous chemical collection from the Kurz-laboratory [19].

### 2.2. Inhibitory Effects of Active Compounds on Larval Motility and Development

**Figure 2:**
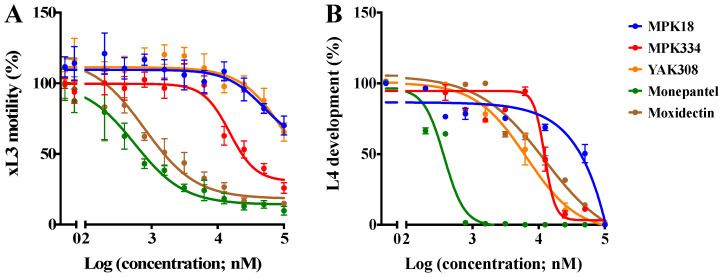
In vitro-activity of three active compounds against exsheathed third-stage larvae (xL3) of *Haemonchus contortus*. Dose-response curves of the individual compounds (MPK18, MPK334 and YAK308) and two control compounds (monepantel and moxidectin) assessing the inhibition of xL3-motility at 72 h (**panel A**) and larval development over seven days (**panel B**). Data points represent three independent experiments conducted in triplicates; the mean ± standard error of the mean (SEM).

**Figure 3:**
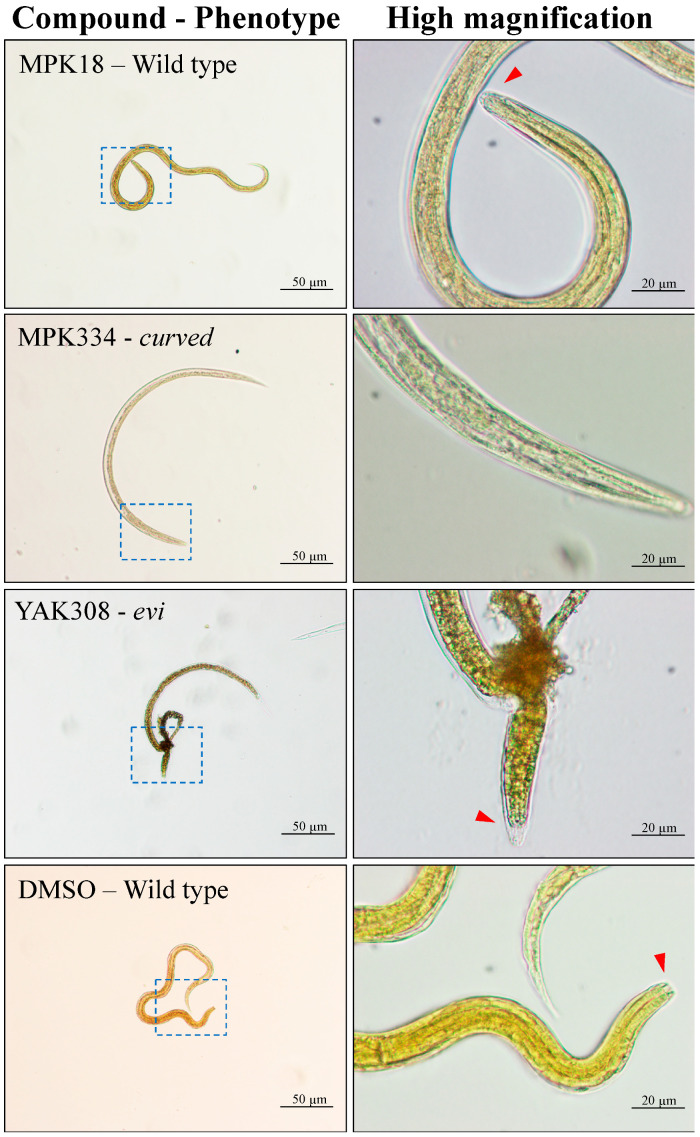
Micrographs of exsheathed third-stage larvae (xL3s) or fourth-stage larvae (L4s) of *Haemonchus contortus* exhibiting different phenotypes after exposure for 7 days to compounds (MPK18, MPK334 or YAK308) or negative control (DMSO). The representative larvae were imaged following the exposure to each of compounds at 100 µM over seven days. A section of each micrograph (blue dashed-box) under 20-times magnification (left panels) were viewed under a higher magnification (100-times magnification; right panels) for the detailed anterior region of individual larva. The L4s have a developed mouth (red arrow).

The three active compounds (MPK18, MPK334 and YAK308) were subsequently assessed for their potencies in a dose-response assay by measuring the inhibition level on larval motility at 72 h and larval development after seven days. MPK334 was the most potent compound at inhibiting xL3 motility after 72 h, with an IC_50_ value of 17.1 ± 4.2 µM (Table 1). MPK18 and YAK308 also inhibited xL3 motility, but less than MPK334 (IC_50_ = 45.2 ± 4.6 µM and IC_50_ = 52.7 ± 6.7 µM; Table 1, respectively). MPK18 and YAK308 followed the same inhibitory trend, despite belonging to different chemotypes (Figure 2). Furthermore, all three compounds inhibited larval development over the seven-day incubation period, with YAK308 being most potent (IC_50_ = 6.5 ± 1.6 µM; Table 1), followed by MPK334 (IC_50_ = 12.3 ± 2.3 µM; Table 1) and MPK18 (IC_50_ not determined; active at >25 µM; Figure 2). Interestingly, MPK334 induced a curved phenotype at 100 µM in 100% of xL3s (Figure 3), even though a coiled phenotype was observed at 20 µM in the primary screen. Similarly, YAK308 induced an “evisceration” phenotype at 100 µM in 22% of xL3s and L4s (Figure 3), while it induced a curved phenotype in the primary screen. This alteration in phenotype was not observed for MPK18, despite its effect on xL3s in the primary screen.

Compound MPK334, representing a chalcone scaffold (1,3-diaryl-2-propen-1-one), was the most potent hit identified in this collection. It was the only compound to inhibit both larval motility and development at low micromolar concentrations, with the level of larval development being similar to the positive–control, moxidectin. The elucidation of the molecular mechanism(s) causing the coiled and curved phenotypes observed in the MPK334-treated xL3s could help understand the mode(s) of action of chalcones against *H. contortus*. The chalcones are known to exert adverse effects on parasitic helminths, including schistosomes and plant nematodes [20,21,22] as well as protists such as *Plasmodium* [23], *Giardia* and *Leishmania* [24,25,26]; however, the exact mode(s) of action in parasites is unknown. The chalcone is proposed to induce pathological changes in musculature in *H. contortus* xL3s by causing cell dysfunction via the inhibition of tyrosine kinase and/or microtubule formation [27], although such effects have only been observed in mammalian systems—in vitro (mammalian cells) and in vivo (murine models) [28]. Some of the unique biological activities of known chalcones are associated with their Michael acceptor features [28,29,30]. This aspect warrants further investigation.

The thiazole–derivative, YAK308 (2-amino-4-phenylthiazole), was potent against *H. contortus*, having a remarkable inhibition of larval development at lower concentrations than for moxidectin. YAK308 induced a lethal evisceration phenotype with a protrusion of the alimentary tract and surrounding tissues through the excretory pore, mainly in the L4 stage at 100 µM, and a curved phenotype at 20 µM in xL3s. Light microscopic examination of L4s at high magnification confirmed the location of visceral extrusion and suggesting that this phenotype relates to a dysfunctional excretory/secretory system and/or cuticle shedding process required for ecdysis (cf. [31,32]), which is crucial for developmental transition. Previously, selected thiazole–derivatives have shown some potential as anthelmintics [33], but this particular 2-amino-4-phenylthiazole, with two methoxy groups, is unique and had not been tested before against a parasitic nematode. Thus, given its potency, further exploration of this scaffold is warranted.

A chalcone, designated MPK318 (Supplementary Table S1)—a close analogue of MPK334 with no 4-bromo substituent—was inactive against xL3s, despite sharing the same reactivity (Michael acceptors). Although not a chalcone, MPK18 is also a Michael acceptor, but it is less potent than MPK334 and it also lacks a 4-bromo substituent. Thus, the 4-substitution might relate to potency. Similarly, compounds BLK133, BLK136 and BLK155 (Supplementary Table S1), which are structurally akin to YAK308 with 5-carbonyl or 5-methyl hydroxy substitution, did not show activity in the primary screen on xL3s. This information suggests that the carbonyl at 5-position may be detrimental to activity of the thiazole structural class.

Initial assessments on HepG2 human hepatoma cells did not reveal toxicity of any of the three hit compounds at concentrations of ≤100 µM, which agrees with previous findings indicating that chalcones and thiazole–derivatives are typically well-tolerated by mammals [29]. The selectivity indices of three hit compounds ranged from 1.9 to 16.1 (Table 1); the highest selectivity index was observed for YAK308 for developmental inhibition of the L4 stage. Cell viability was 100% for 100 µM, suggesting that the true selectivity index might be even greater. Thus, these compounds/scaffolds could have potential for optimisation to achieve potencies in the sub-micromolar range. Further modifications of MPK334, such as synthetic hybrids with a benzimidazolyl group, could potentially increase its anthelmintic activity [34]. Conjugation of an additional moiety on YAK308 may increase potency, as has been demonstrated for other anti-parasitic (e.g., aryl-thiazole derivatives) [33] and anti-inflammatory drugs (e.g., benzimidazole-thiazole hybrids) [35]. The newly synthesised analogues would need to be re-tested in vitro against larvae and then on *H. contortus* adults—which are responsible for haemonchosis in ruminant hosts and are the target for treatment in the stomach (i.e., abomasum).

## 3. Materials and Methods

### 3.1. Compound Preparation for Screening

A library containing 245 compounds (Supplementary Table S1) was curated by the authors (T.K. and B.L.) at the Institute of Pharmaceutical and Medicinal Chemistry, Heinrich-Heine-University Düsseldorf, Germany. Individual compounds were dissolved in 100% high-performance liquid chromatography-grade dimethyl sulfoxide (DMSO; Merck, USA) to achieve stock concentrations of 20 mM. For screening, individual compounds were diluted further to the final concentration of 20 µM in Luria Bertani broth (LB) supplemented with 100 U/mL of penicillin, 100 μg/mL of streptomycin and 0.25 μg/mL of amphotericin B (designated LB*), and tested for activity in vitro against *H. contortus*.

### 3.2. Procurement and Preparation of Parasite Larvae

*Haemonchus contortus* (Haecon-5 strain) was maintained in experimental sheep [36], in accordance with institutional animal ethics approval (permit no. 1714374; The University of Melbourne). Helminth-free Merino sheep (male; eight months of age) were inoculated orally with 7000 third-stage larvae (L3s) of *H. contortus*. Faecal samples containing *H. contortus* eggs were collected every day from 21 days following inoculation. These samples were incubated at 27 °C for one week to produce L3s [14], which were harvested and sieved through two layers of nylon mesh (20 μM pore size; Rowe Scientific, Australia) to remove debris and dead larvae, and then stored at 11 °C for up to 6 months until use. To produce exsheathed third-stage larvae (xL3s) for in vitro assays, L3s were incubated in 0.15% (*v/v*) sodium hypochlorite for 20 min at 37 °C followed by five washes in 0.9% sterile saline by centrifugation at 600× *g* (5 min) at room temperature (22–24 °C) [14].

### 3.3. Screening of Compounds on H. contortus xL3s

Compounds were screened at 20 μM on *H. contortus* xL3s using an established assay [14]. In brief, diluted test-compounds, negative controls (LB* + 0.5% DMSO) and two positive controls [20 μM of monepantel (Zolvix, Novartis Animal Health, Switzerland) and 20 μM of moxidectin (Cydectin, Virbac, France)] were added to wells of the 96-well microtiter plates (Corning, USA), and xL3s were dispensed at ~300/well to a final volume of 100 µL. Following a 72 h-incubation at 38 °C and 10% (*v*/*v*) CO_2_ at >90% humidity, a video recording (5 s) was taken of each well of the 96-well microtitre plate containing xL3s using a gray–scale camera (Rolera Bolt Scientific CMOS, Teledyne QImaging, Tucson, AZ, USA) and a motorised X–Y axis stage (BioPoint 2, Ludl Electronics Products, Hawthorne, CA, USA) attached to a stereomicroscope (SZ61, Olympus, Shinjuku, Japan). Primary screening was performed in duplicate. A compound was recorded as having activity if it altered worm morphology (phenotype) after 72 h of incubation.

### 3.4. Assessing Inhibitory Activity on Larval Motility

Three compounds (designated MPK18, MPK334 and YAK308) shown to have activity against *H. contortus* in the primary screen were evaluated for their potency by estimating their half maximal inhibitory concentrations (IC_50_), calculated in a dose-response assay. In brief, compounds were titrated (18-points, 2-fold serial dilution in 50 μL of LB*, 100 µM down to 0.76 nM), in 96-well microtitre plates and xL3s were dispensed in 50 µL at a density of ~300 per well. All plates were incubated at 38 °C and 10% (*v/v*) CO_2_ with >90% humidity. After 72 h of incubation, a video recording (5 s) was taken of each well in the same camera settings as the primary screen (see Section 3.3), and larval motility measured. The motility index (Mi) was calculated from video data using the unique algorithm written in a custom macro, and analysed through the program ImageJ (v.2.0.0, Fiji) [14]. Mi data were normalised to a percentage compared with negative control (LB* + 0.5% DMSO). Monepantel and moxidectin were diluted in the same manner, and used as positive control compounds. Assays were performed in triplicate, three times, on separate days. To establish IC_50_ values, compound concentrations were log_10_-transformed, fitted using a variable slope four-parameter equation, constraining the maximum value to 100% using a least squares (ordinary) fit model employing GraphPad Prism (v. 9.0.2) software.

### 3.5. Evaluating Inhibitory Activity on Larval Development

Following the measurement of larval motility at 72 h, plates containing xL3s were incubated for four more days at 38 °C [10% (*v/v*) CO_2_ and >90% relative humidity] to test the active compounds (MPK18, MPK334 and YAK308) for their effect on the larval development over 7 days. Subsequently, worms were fixed in 1% iodine, and the rate of larval development was assessed microscopically appraising the presence (L4) or absence (xL3) of mouth and pharynx [37], and direct comparison with negative (untreated) controls (LB* + 0.5% DMSO). Thirty worms from each well were examined at 50-times magnification, and the number of L4s was expressed as a percentage of the total number. Assays were performed in triplicate, three times, on separate days. To establish IC_50_ values, compound concentrations were log_10_-transformed, fitted using a variable slope four-parameter equation with constraining the top value to 100% using a least squares (ordinary) fit model using GraphPad Prism (v. 9.0.2) software.

### 3.6. Evaluation of Toxicity of Active Compounds on HepG2 Cells

HepG2 human hepatoma cells were seeded into wells of a 96-well microtitre plate in 80 µL of Dulbecco’s modified eagle medium (DMEM), supplemented with 10% foetal bovine serum, 100 U/mL of penicillin, 100 µg/mL of streptomycin and 0.25 µg/mL of amphotericin B (DMEM*) at a density of 1 × 10^5^ cells per well. Cells were allowed to adhere for 16 h at 37 °C and 5% (*v/v*) CO_2_ at >90% humidity, and incubated with individual, serially-diluted compounds (7-points, 2-fold dilution in DMEM*, from 100 µM to 1.56 µM) in a final volume of 100 µL. Doxorubicin (10 µM) and DMEM* + 0.25% DMSO (vehicle), serving as positive and negative controls, respectively, were added to the individual 96-well plates. Monepantel and moxidectin were also serially diluted—prepared in the same manner as active compounds—and used as ‘negative’ reference controls. After 48 h of incubation, cellular viability was determined by the crystal violet staining assay [38]. The absorbance at 595 nm of treated cells was compared with that of the controls (exposed only to the vehicle, with 100% viability). Compounds and controls were tested in triplicate. To determine the half cytotoxic concentration (CC_50_) values, compound concentrations were log_10_-transformed, fitted using a variable slope four-parameter equation with a least squares (ordinary) fit model employing GraphPad Prism (v. 9.0.2) software.

## 4. Conclusions

The findings for the three new chemical entities with in vitro-anthelmintic activities against *H. contortus* larvae encourage future SAR studies of analogues of MPK18, MPK334 and YAK308, with a future focus on developing an anthelmintic against strongylid nematodes of animals.

## Figures and Tables

**Table 1:** In vitro-activities of three hit compounds (MPK18, MPK334 and YAK308) against exsheathed third-stage larvae (xL3) of *Haemonchus contortus*. Half maximal inhibitory concentration (IC_50_) values of each compound on the larval motility after 72 h incubation and the larval development into fourth-stage larvae (L4) after seven days are presented in reference to the values of two control compounds (monepantel and moxidectin) obtained in the same assay condition. Half cytotoxic concentration (CC_50_) values against HepG2 human hepatoma at 48 h are presented for five test and control compounds. The selectivity indices (SI) were calculated for five compounds based on their IC_50_ and CC_50_ values. The IC_50_ values presented are the mean ± standard error of the mean (SEM). Three independent experiments were conducted in triplicates.

Compound	xL3 Motility (72 h) IC_50_ ± SEM (µM)	xL3 Motility (72 h) Inhibition (%)	L4 Development (7 days) IC_50_ ± SEM (µM)	HepG2 Cells (48 h) CC_50_ (µM)	SI xL3 Motility/L4 Development
MPK18	45.2 ± 4.6	30.0	nd	>100	>1.0/nd
MPK334	17.1 ± 4.2	74.2	12.3 ± 2.3	>100	5.8/8.1
YAK308	52.7 ± 6.7	34.6	6.5 ± 1.6	>100	>1.0/16.1
Monepantel	0.6 ± 0.1	90.3	0.4 ± 0.05	>100	166/250
Moxidectin	0.8 ± 0.2	85.1	12.4 ± 0.1	>100	125/8.1

Not determined (nd).

## Supplementary Materials

The following are available online, Table S1: The 245 synthetic compounds from the Kurz collection screened in this study.
