# Supplementary material for: Three Small Molecule Entities (MPK18, MPK334 and YAK308) with Activity against Haemonchus contortus In Vitro
Source: Molecules. 2021 May 10;26(9):2819. doi: 10.3390/molecules26092819 (PMC8126080; doi:10.3390/molecules26092819)
Supplement: Supplementary file 1 [file molecules-26-02819-s001.zip › molecules-1175734-SI.pdf]

Supplementary File

Three small molecule entities (MPK18, MPK334 and YAK308) with activity against *Haemonchus contortus* in vitro

Aya C. Taki [1], Abdul Jabbar [1], Thomas Kurz [2], Beate Lungerich [2], Guangxu Ma [1], Joseph J. Byrne [1], Marc Pflieger [2], Yodita Asfaha [2], Fabian Fischer [2], Bill C. H. Chang [1], Brad E. Sleebs [1,3,4], Robin B. Gasser [1,*]

[1] Department of Biosciences, Melbourne Veterinary School, Faculty of Veterinary and Agricultural Sciences, The University of Melbourne, Parkville, Victoria, Australia; aya.taki@unimelb.edu.au (A.C.T.); jabbara@unimelb.edu.au (A.J.); guangxu.ma@unimelb.edu.au (G.M.); bchang@yourgene.com.tw (B.C.H.C.); sleebs@wehi.edu.au (B.E.S.)

[2] Institute of Pharmaceutical and Medicinal Chemistry, Heinrich-Heine-University Düsseldorf, Düsseldorf, Germany; thomas.kurz@hhu.de (T.K.); beate.lungerich@hhu.de (B.L.); pflieger@hhu.de (M.P.); yodita.asfaha@uni-duesseldorf.de (Y.A.); Fabian.Fischer.14@uni-duesseldorf.de (F.F.)

[3] Walter and Eliza Hall Institute of Medical Research, Parkville, Victoria, Australia;

[4] Faculty of Medicine, Dentistry and Health Sciences, The University of Melbourne, Parkville, Victoria, Australia

* Correspondence: robinbg@unimelb.edu.au (R.B.G.)

Table S1. The 245 synthetic compounds from the Kurz collection screened in this study.

Name	Structure	molecular weight [g/mol]	Chemotype
VWK135	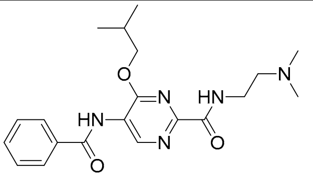	385,46	4-alkoxypyrimidine-2-carboxamide
VWK176	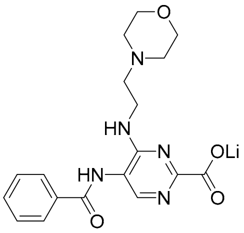	377,33	Lithium 4-(alkylamino)pyrimidine-2-carboxylate
VWK177	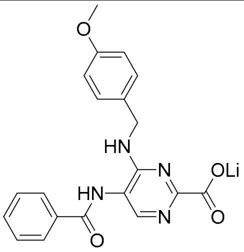	384,32	Lithium 4-(arylmethylamino)pyrimidine-2-carboxylate
VWK183	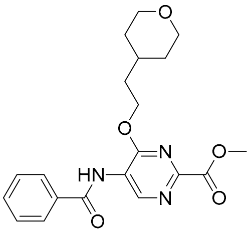	385,42	methyl 4-alkoxypyrimidine-2-carboxylate
VWK189	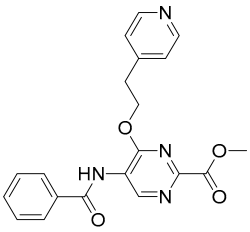	378,39	methyl 4-alkoxypyrimidine-2-carboxylate
VWK202	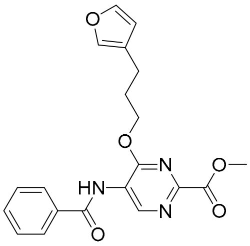	381,39	methyl 4-alkoxypyrimidine-2-carboxylate

VWK205	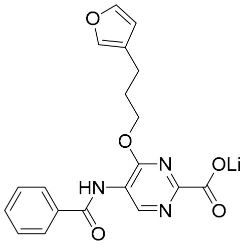	373,29	Lithium 4-alkoxypyrimidine-2-carboxylate
VWK240	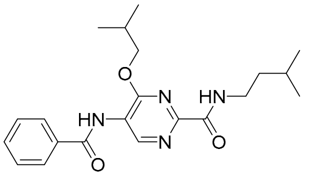	384,48	4-alkoxypyrimidine-2-carboxamide
VWK244	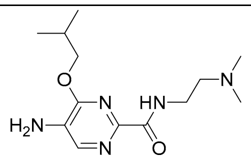	281,36	4-alkoxypyrimidine-2-carboxamide
VWK277	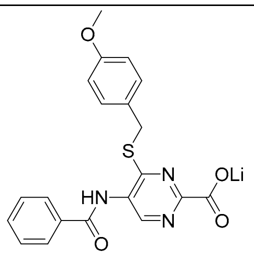	401,37	Lithium 4-(arylmethylamino)pyrimidine-2-carboxylate
VWK292	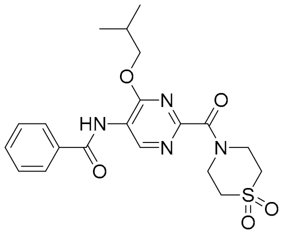	432,5	4-alkoxypyrimidine-2-carboxamide
VWK329	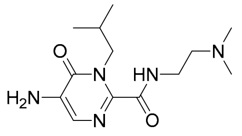	281,36	5-amino-1-alkylyl-6-oxo-1,6-dihydropyrimidine-2-carboxamide
VWK356	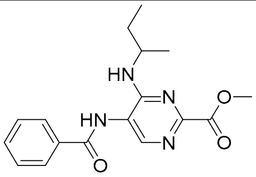	328,37	methyl 4-(alkylamino)pyrimidine-2-carboxylate
VWK375	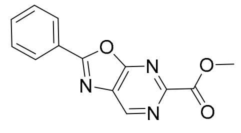	255,23	methyl oxazolo[5,4-d]pyrimidine-5-carboxylate

VWK382	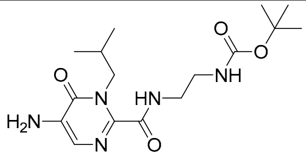	353,42	5-amino-1-alkylyl-6-oxo-1,6-dihydropyrimidine-2-carboxamide
VWK383	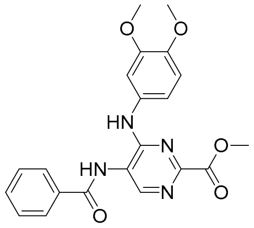	408,41	methyl 4-(arylmethylamino)pyrimidine-2-carboxylate
VWK395	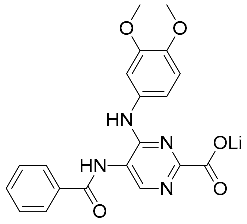	400,32	Lithium 4-(arylamino)pyrimidine-2-carboxylate
VWK397	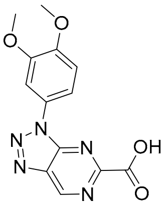	301,26	3-(aryl)-3H-[1,2,3]triazolo[4,5-d]pyrimidine-5-carboxylic acid
VWK401	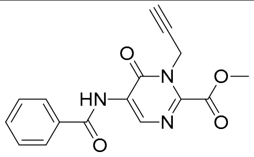	311,3	methyl 1-alkyl-6-oxo-1,6-dihydropyrimidine-2-carboxylate
VWK412	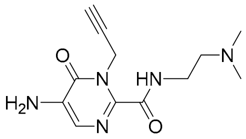	263,3	5-amino-1-alkylyl-6-oxo-1,6-dihydropyrimidine-2-carboxamide
VWK418	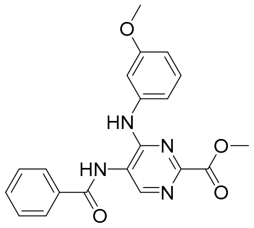	378,39	methyl 4-(arylamino)pyrimidine-2-carboxylate
VWK419	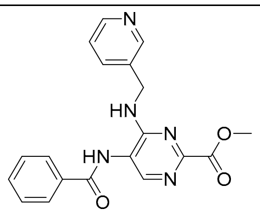	363,38	methyl 4-(arylmethylamino)pyrimidine-2-carboxylate

VWK421		432,36	methyl 4-(arylamino)pyrimidine-2-carboxylate
VWK426		368,42	methyl 4-(arylmethylamino)pyrimidine-2-carboxylate
VWK427-N		347,39	methyl 1-alkyl-6-oxo-1,6-dihydropyrimidine-2-carboxylate
VWK427-O		347,39	methyl 4-alkoxypyrimidine-2-carboxylate
VWK428		444,49	methyl 4-alkoxypyrimidine-2-carboxylate
VWK430		387,39	methyl 4-alkoxypyrimidine-2-carboxylate
BLK104	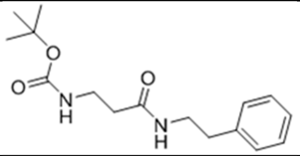	292,38	3-aminopropanamide
BLK110	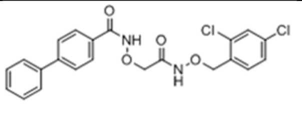	445,3	2-(aminooxy)-*N*-hydroxyacetamide

BLK115	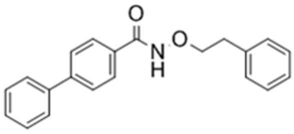	317,39	N-(Allkoxy)benzamide
BLK116	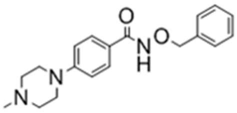	325,41	N-(Aralkoxy)benzamide
BLK120	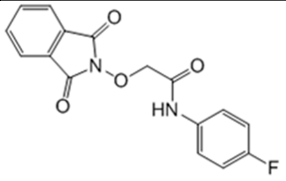	314,27	2-(aminooxy)acetamide
BLK122	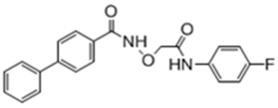	364,38	2-(aminooxy)acetamide
BLK126	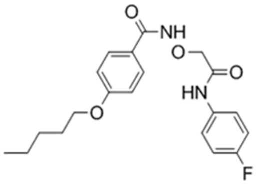	374,41	2-(aminooxy)acetamide
BLK129	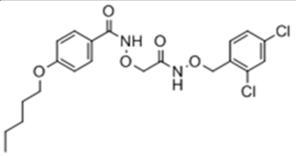	455,33	2-(aminooxy)-N-hydroxyacetamide
BLK132	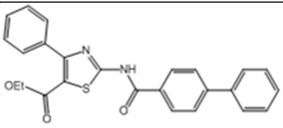	428,51	2-amino-4-phenylthiazole
BLK133	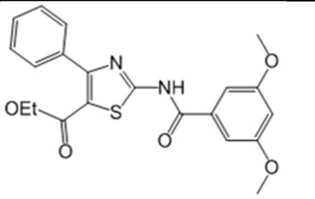	412,46	2-amino-4-phenylthiazole
BLK136	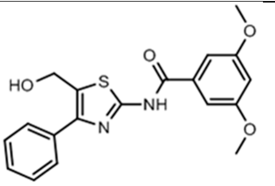	370,42	2-amino-4-phenylthiazole
BLK137	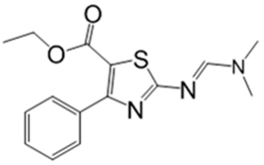	303,38	2-amino-4-phenylthiazole

BLK154	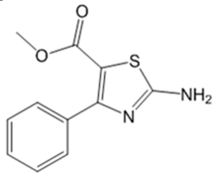	234,27	2-amino-4-phenylthiazole
BLK155	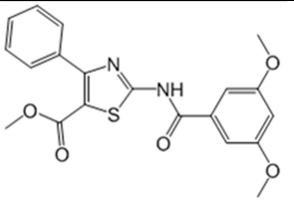	398,43	2-amino-4-phenylthiazole
BLK156	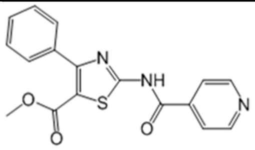	339,37	2-amino-4-phenylthiazole
BLK163	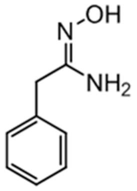	150,18	(Z)-*N'*-hydroxy-2-phenylacetimidamide
BLK166	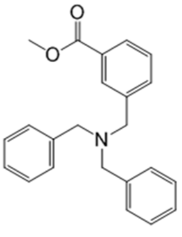	345,44	tribenzylamine
BLK170	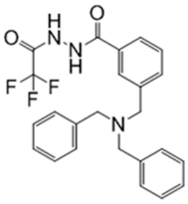	441,45	tribenzylamine
BLK171	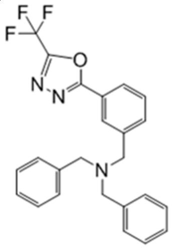	423,44	tribenzylamine
BLK172	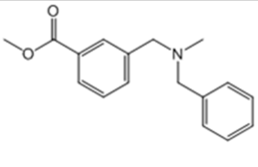	269,34	dibenzylmethlamine

BLK174	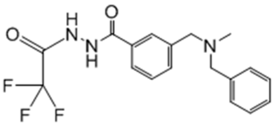	365,36	*N*-benzyl-*N*-methyl-1-phenylmethanamine
BLK175	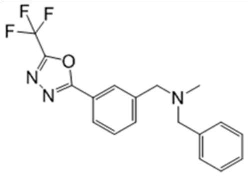	347,34	*N*-benzyl-N-methyl-1-phenylmethanamine
BLK177	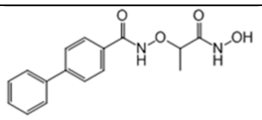	300,31	2-(aminooxy)-N-hydroxyacetamide
BLK178	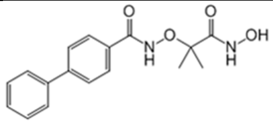	314,3	2-(aminooxy)-N-hydroxyacetamide
BLK183	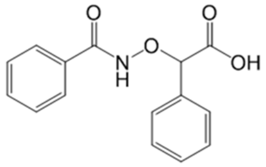	271,3	2-(aminooxy)-2-phenylacetic acid
BLK188		347,37	2-(aminooxy)-2-phenylacetic acid
BLK189	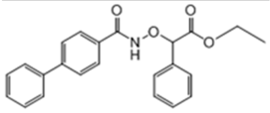	375,4	2-(aminooxy)-2-phenylacetic acid
BLK191	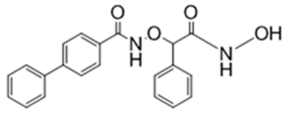	362,4	2-(aminooxy)-*N*-hydroxy-2-phenylacetamide
BLK194	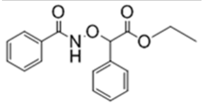	299,3	2-(aminooxy)-2-phenylacetic acid
BLK195	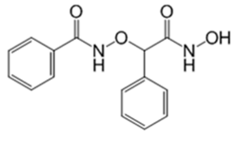	286,3	2-(aminooxy)-*N*-hydroxy-2-phenylacetamide
MLKQnP1	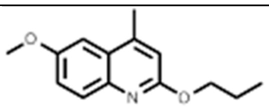	231,3	Quinoline

MLKQ9	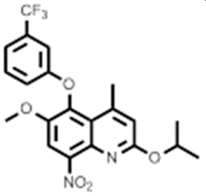	436,39	Quinoline
MLKQiP2	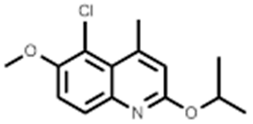	265,74	Quinoline
MLKQE3	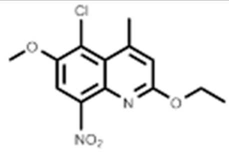	296,71	Quinoline
MLKQnP2	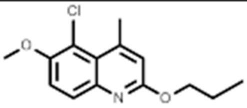	265,74	Quinoline
MLKQ5	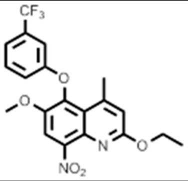	422,36	Quinoline
MLKQ6	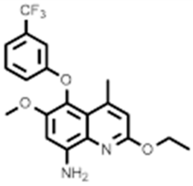	392,38	Quinoline
MLKd1a	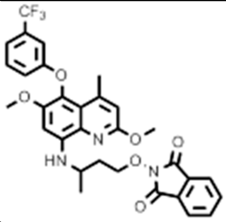	595,58	Quinoline
MLKh1a	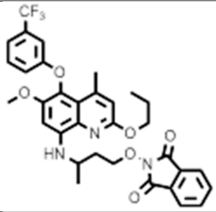	623,63	Quinoline
TKK107		397.54	hydrazonamide
TKK108		480.7	hydrazonamide
TKK119		325.46	hydrazonamide
N1-BA-AAH		283,38	hydrazonamide
N1-4OMe-AAH		347,42	hydrazonamide

N1-4diMeN-AAH	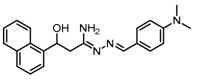	360,46	hydrazonamide
N1-ICA-AAH	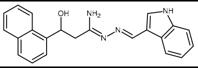	356,43	hydrazonamide
N1-F2CA-AAH	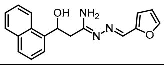	307,35	hydrazonamide
N1-4triFMe-AAH	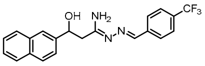	385.39	hydrazonamide
N1-34diCl-AAH	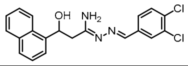	386.28	hydrazonamide
N1-4diEtNMe-AAH	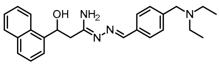	402.54	hydrazonamide
N1-PA-AAH	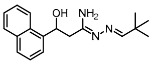	297.40	hydrazonamide
N2-4triFMe-AAH	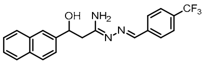	385.39	hydrazonamide
N2-4Me-AAH	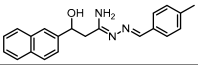	331.42	hydrazonamide
N2-24diF-AAH	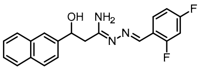	353.37	hydrazonamide
N2-4OMe-AAH	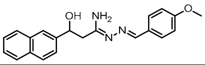	347.42	hydrazonamide
N2-P3CA-AAH	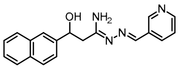	318.38	hydrazonamide
N2-34diCl-AAH	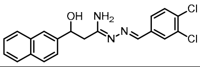	386.28	hydrazonamide
N2-3OEt4OH-AAH	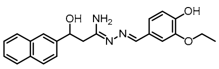	377.44	hydrazonamide
N2-4OBn-AAH	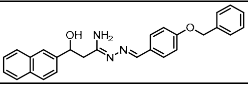	423,52	hydrazonamide
N2a-AAH	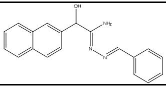	303,37	hydrazonamide
P9-5NA-AAH	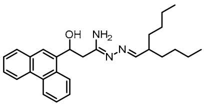	417,60	hydrazonamide
P9-4F-AAH	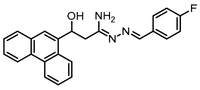	385,44	hydrazonamide
P9-Cyh-AAH	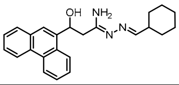	373,50	hydrazonamide
BLK003	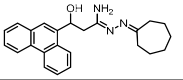	373,50	hydrazonamide
BLK001	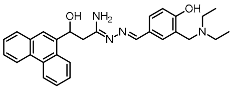	468,60	hydrazonamide
BLK004	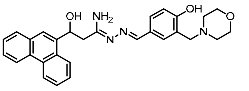	482,58	hydrazonamide
BLK010	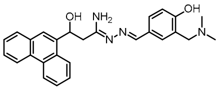	440,55	hydrazonamide

BLK019	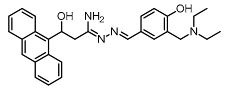	468,60	hydrazonamide
BLK021	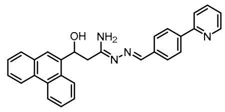	444,54	hydrazonamide
BLK023	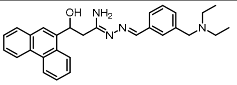	452,60	hydrazonamide
BLK080	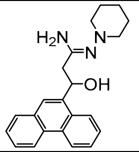	347,46	hydrazonamide
BLK081	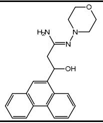	349,43	hydrazonamide
BLK082	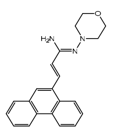	331,42	hydrazonamide
BLK084	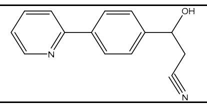	224,26	arylalcohol
BLK086	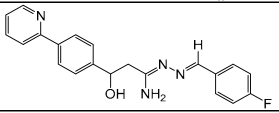	362,41	hydrazonamide
ABK-377	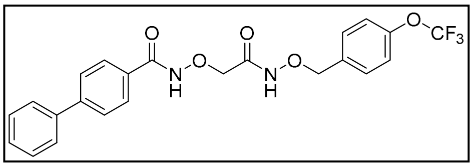	460,41	2-(aminooxy)-*N*-hydroxyacetamide
ABK-394	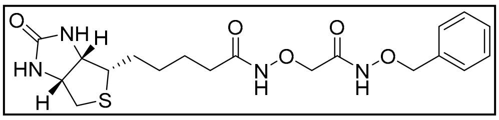	422,51	2-(aminooxy)-*N*-hydroxyacetamide
ABK-403	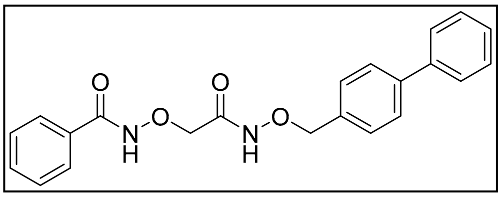	376,41	2-(aminooxy)-*N*-hydroxyacetamide
ABK-421	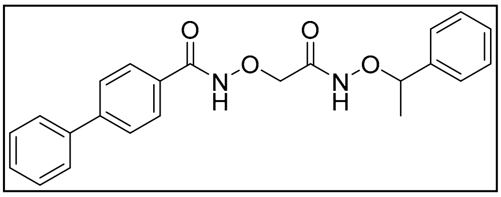	390,44	2-(aminooxy)-*N*-hydroxyacetamide

ABK-422	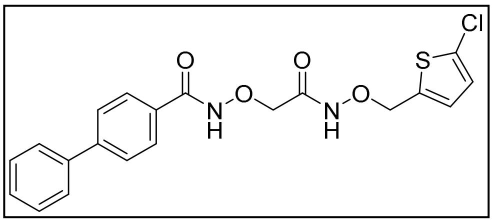	416,88	2-(aminooxy)-*N*-hydroxyacetamide
MPK142	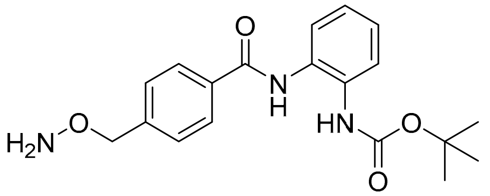	357.41	(2-(4-((aminooxy)methyl)benzamido)phenyl
MPK18	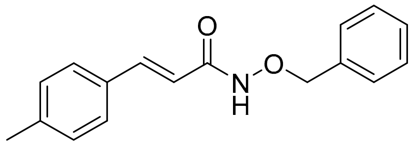	267.33	3-(p-tolyl)acrylamide
MPK96	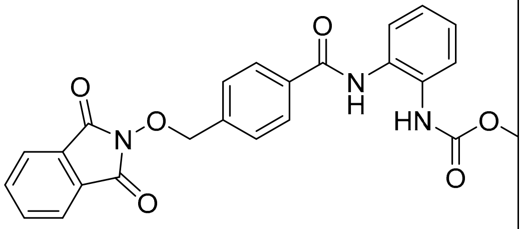	487.51	(2-(4-((aminooxy)methyl)benzamido)phenyl
MPK67	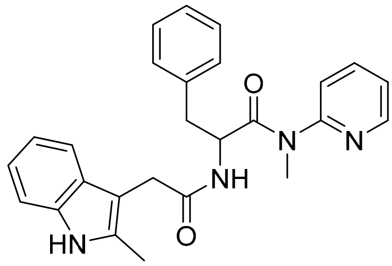	426.52	Phenylalanine derivative
MPK86	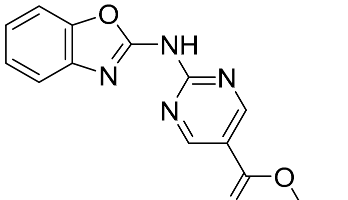	284.28	2-aminopyrimidine
MPK187	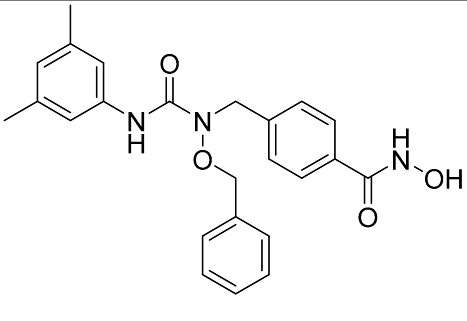	419.48	Hydroxamic acid

MPK192	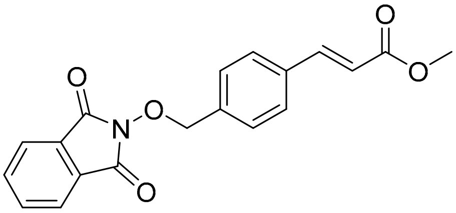	337.33	3-(p-tolyl)acrylate
MPK193	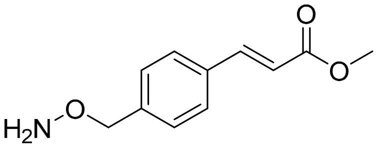	207.23	3-(p-tolyl)acrylate
MPK215	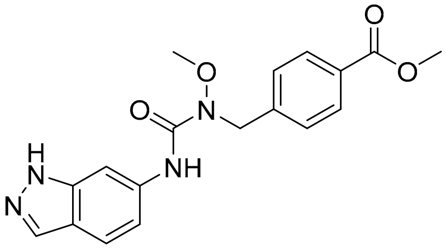	354.37	Methyl benzoate
MPK264	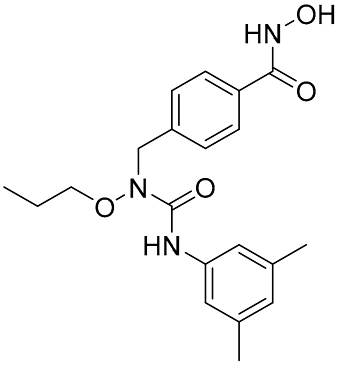	371.44	Hydroxamic acid
MPK265	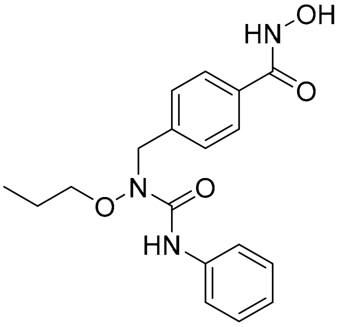	343.38	Hydroxamic acid
MPK318	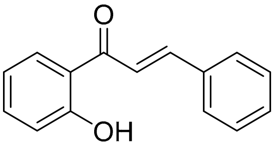	224.26	chalcone
MPK317	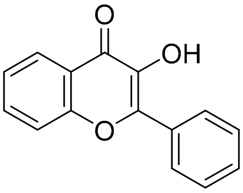	238.24	4*H*-chromen-4-one

MPK329	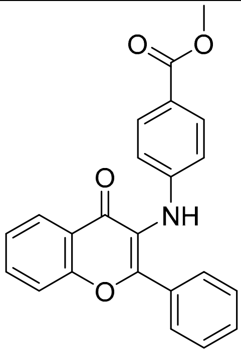	371.39	4*H*-chromen-4-one
MPK324	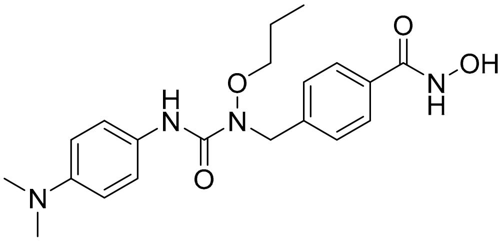	386.45	Hydroxamic acid
MPK328	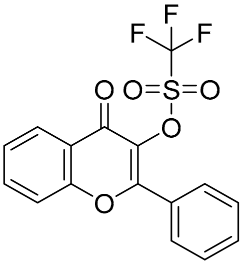	370.30	4*H*-chromen-4-one
MPK334	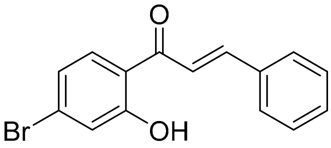	303.16	Chalcone
MPK380	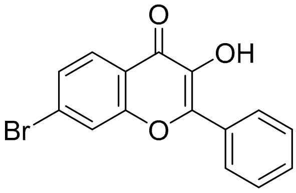	317.14	4*H*-chromen-4-one
MPK377	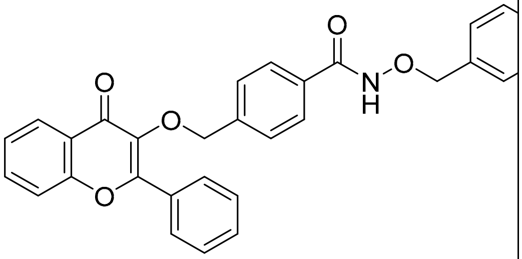	477.52	4*H*-chromen-4-one

MPK375	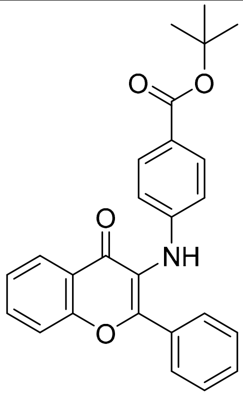	413.47	4*H*-chromen-4-one
MPK391	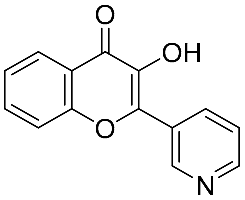	239.23	4*H*-chromen-4-one
MPK396	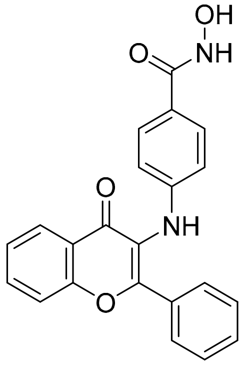	372.38	4*H*-chromen-4-one
MPK406	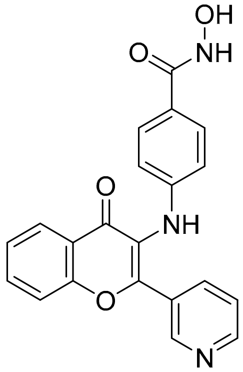	373.37	4*H*-chromen-4-one
MPK409	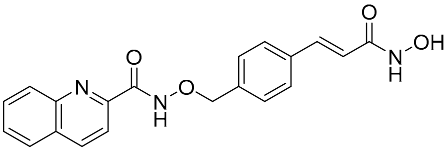	363.37	Hydroxamic acid
MPK414	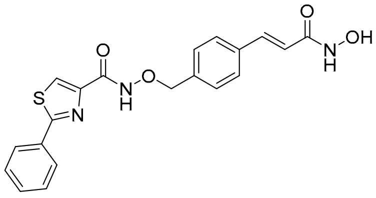	395.43	Hydroxamic acid

MPK415	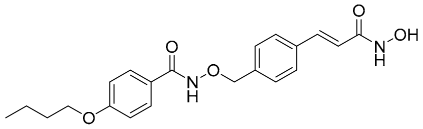	384.43	Hydroxamic acid
MPK416	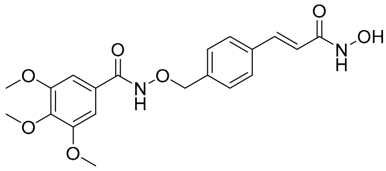	402.40	Hydroxamic acid
MPK421	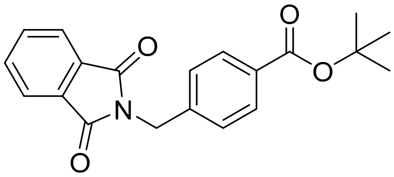	337.38	benzoate
MPK423	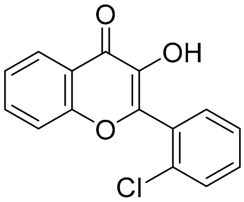	272.68	4*H*-chromen-4-one
MPK428	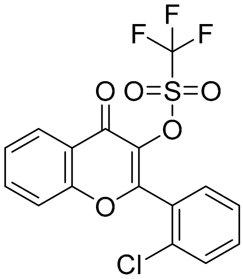	404.74	4*H*-chromen-4-one
MPK439	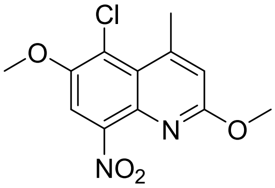	282.68	Quinoline
MPK460	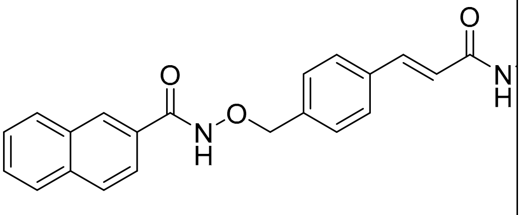	362.39	Hydroxamic acid
MPK461	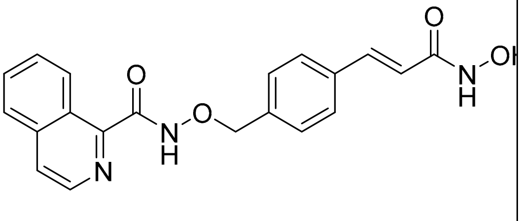	363.37	Hydroxamic acid

MPK472	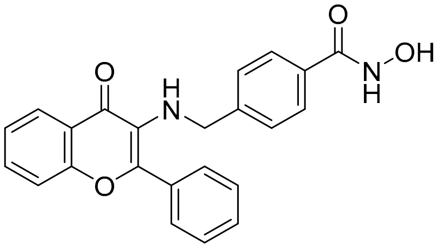	386.41	Hydroxamic acid
MPK481	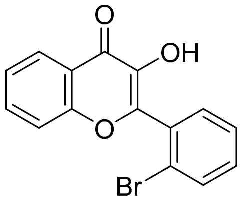	317.14	4*H*-chromen-4-one
MPK490	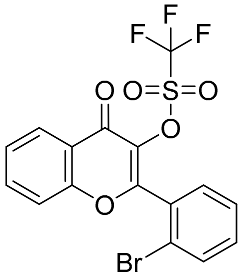	449.19	4*H*-chromen-4-one
MPK508	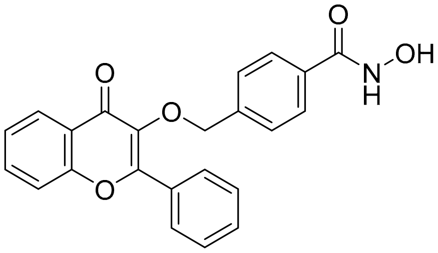	387.39	Hydroxamic acid
MPK509	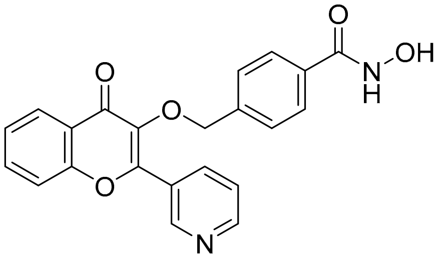	388.38	Hydroxamic acid
MPK511	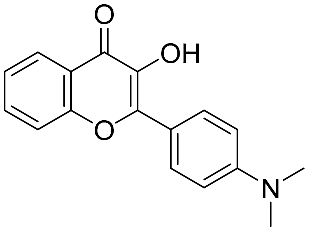	281.31	Hydroxamic acid

MPK522	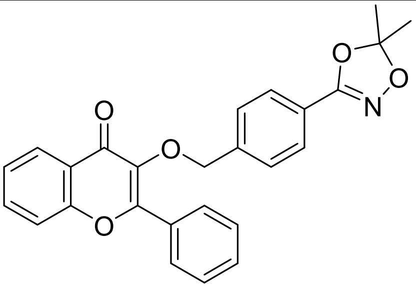	427.46	4*H*-chromen-4-one
MPK525	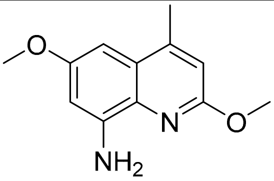	218.26	Quinoline
MPK526	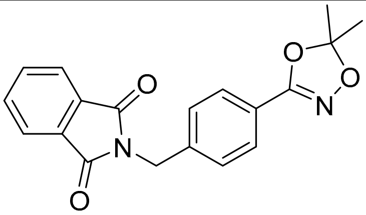	336.35	Dioxazole
MPK541	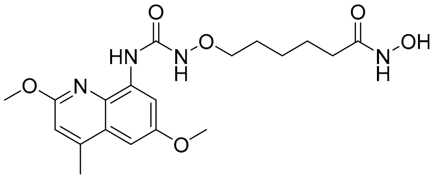	406.44	Hydroxamic acid
MPK542	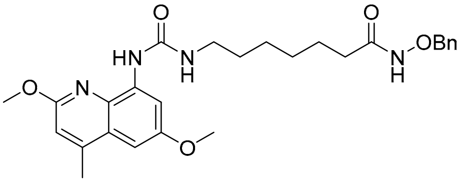	494.59	*O*- protected hydroxamic acid
MPK543	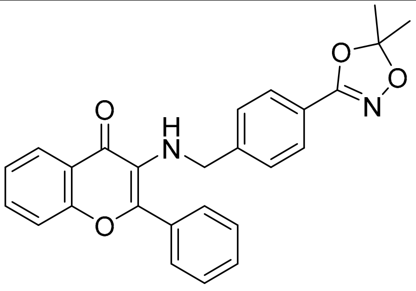	426.47	4*H*-chromen-4-one
MPK544	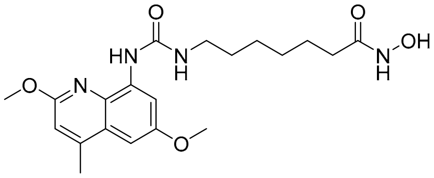	404.47	Hydroxamic acid

MPK545	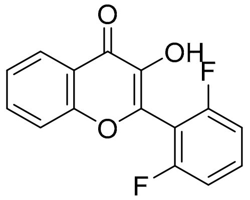	274.22	4*H*-chromen-4-one
MPK546	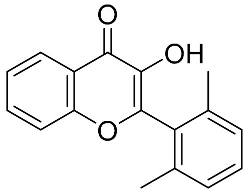	266.30	4*H*-chromen-4-one
MPK557	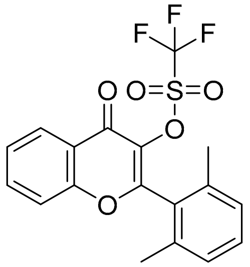	398.35	4*H*-chromen-4-one
MPK558	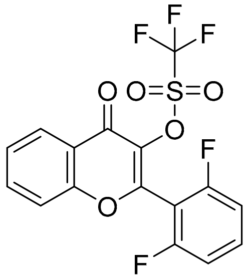	406.28	4*H*-chromen-4-one
MPK563	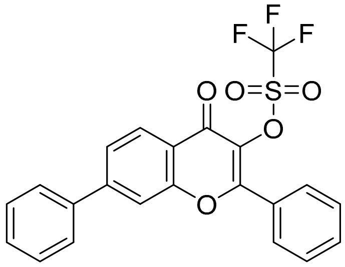	446.40	4*H*-chromen-4-one
MPK567	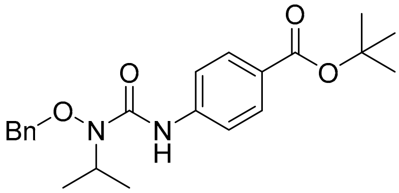	384.48	benzoate
MPK576	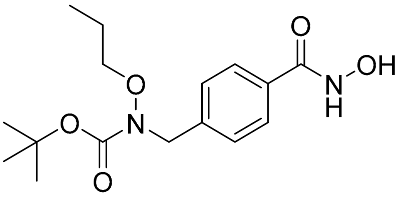	324.38	Hydroxamic acid

LAK4	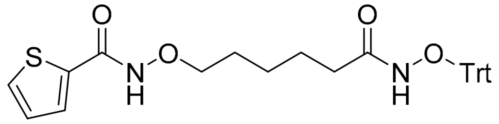	514,64	*O*- protected hydroxamic acid
LAK70	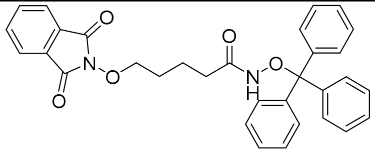	520,58	*O*- protected hydroxamic acid
LAK75	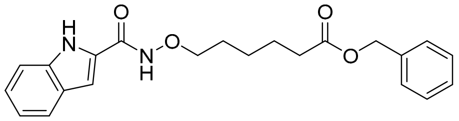	380,44	6-(aminooxy)hexanoic acid
LAK80	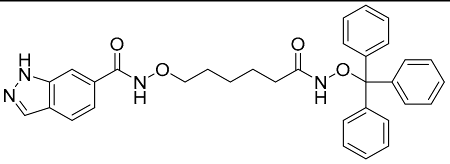	548,64	*O*- protected hydroxamic acid
LAK102	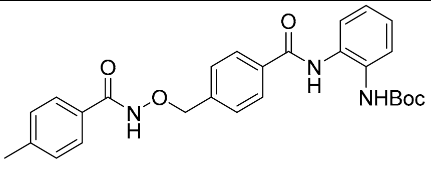	475,54	4-methylbenzamide
LAK104	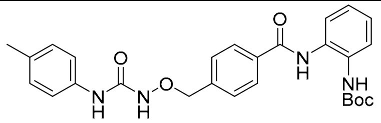	490,56	4-methylbenzamide
YAK 288	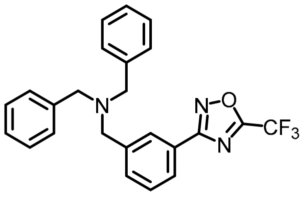	423.44	Trifluoromethyloxadiazole
YAK308	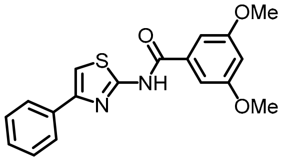	340.40	2-amino-4-phenylthiazole
YAK 312	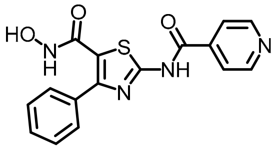	340.36	Hydroxamic acid
YAK325	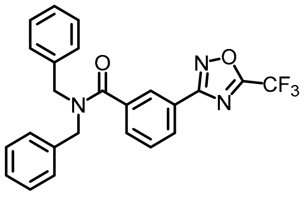	437.42	Trifluoromethyloxadiazole

YAK320	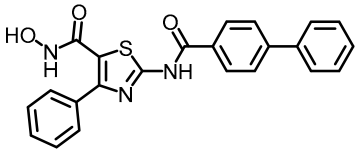	415.47	Hydroxamic acid
YAK 328	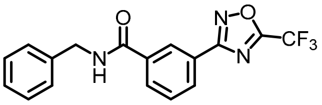	347.30	Trifluoromethyloxadiazole
YAK398	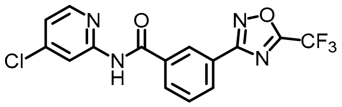	368.70	Trifluoromethyloxadiazole
YSKK82	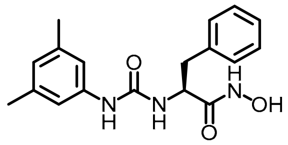	327.38	Hydroxamic acid
YAK 330	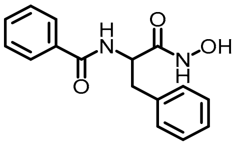	284.32	Hydroxamic acid
YSKK43	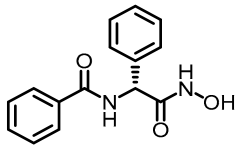	270.29	Hydroxamic acid
YSKK78	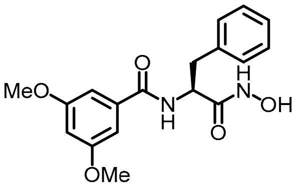	344.37	Hydroxamic acid
YSKK42	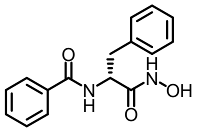	284.32	Hydroxamic acid
YAK375	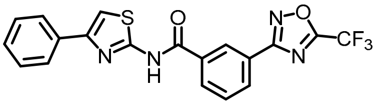	416.38	Trifluoromethyloxadiazole

YAK 383	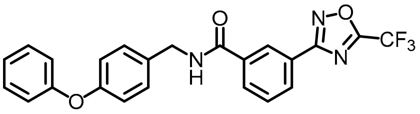	439.39	Trifluoromethyloxadiazole
YAK 378	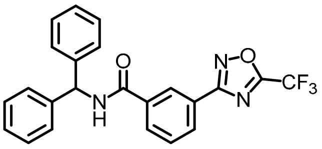	423.40	Trifluoromethyloxadiazole
YAK 384	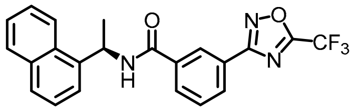	411.38	Trifluoromethyloxadiazole
YAK 376	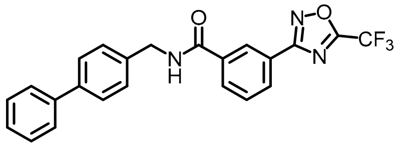	423.40	Trifluoromethyloxadiazole
YAK379	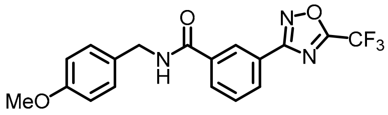	377.32	Trifluoromethyloxadiazole
YAK382	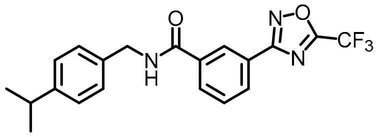	389.38	Trifluoromethyloxadiazole
YAK422	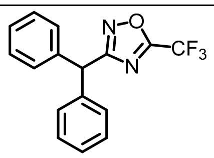	304.27	Trifluoromethyloxadiazole
YAK431	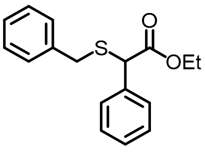 [286.39]	286.39	2-thio-2-phenylacetic acetate

YAK432	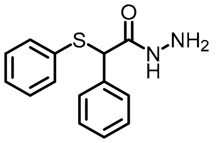 [258.34]	258.34	2-thio-2-phenylacetic acetate
YAK455	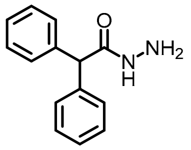 [226.28]	226.28	hydrazide
YAK464	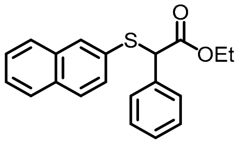 [322.42]	322.42	2-thio-2-phenylacetic acetate
YAK435	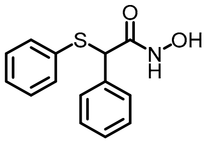	259.32	Hydroxamic acid
YAK440	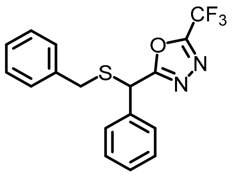	350.36	Trifluoromethyloxadiazole
YAK460	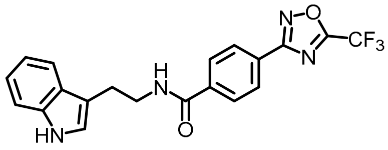	400.36	Trifluoromethyloxadiazole
YAK400	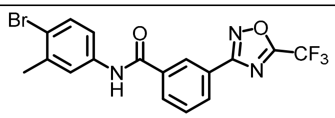	426.19	Trifluoromethyloxadiazole
FFK18	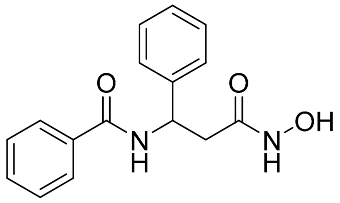	284.32	Hydroxamic acid

FFK19	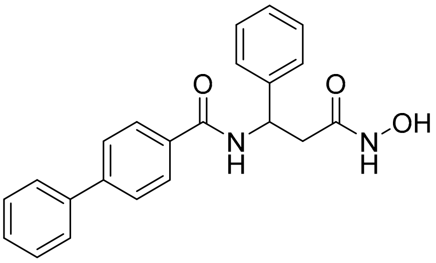	360.41	Hydroxamic acid
FFK22	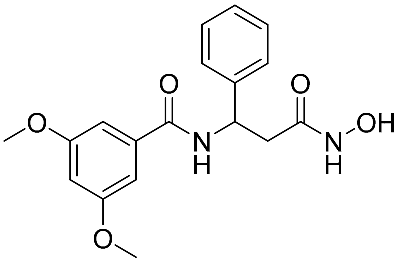	344.37	Hydroxamic acid
FFK23	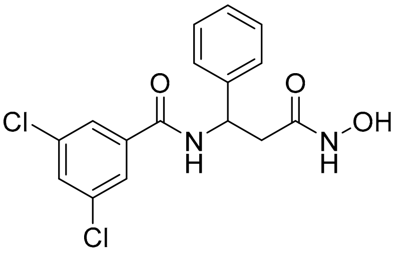	353.20	Hydroxamic acid
FFK33	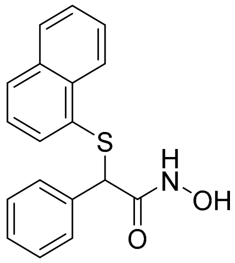	309.38	Hydroxamic acid
YAK468	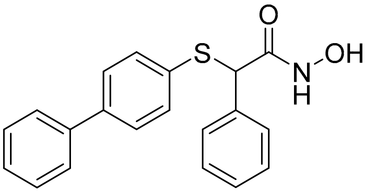	335.43	Hydroxamic acid
YAK448	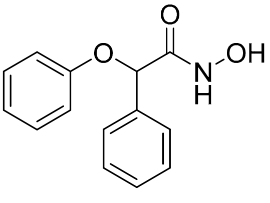	243.26	Hydroxamic acid
YAK439	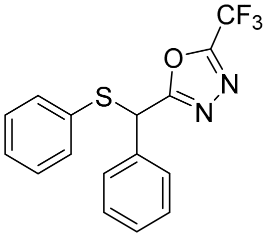	336.34	Trifluoromethyloxadiazole

YAK427	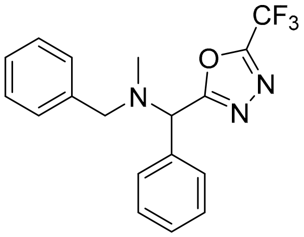	347.34	Trifluoromethyloxadiazole
MPK648	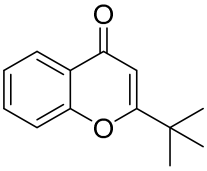	202.25	Flavone
MPK664	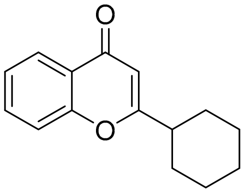	228.29	Flavone
MPK718	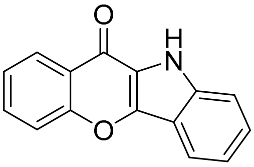	235.24	Chromeno-indol-one
MPK674	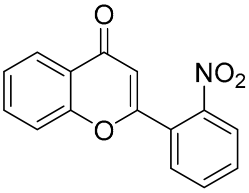	267.24	Flavone
MPK690	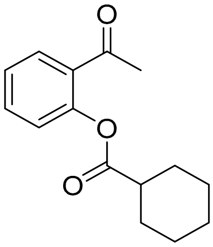	246.31	2´-Hydroxy-acetophenone-ester
MPK691	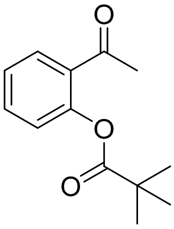	220.27	2´-Hydroxy-acetophenone-ester
MPK694	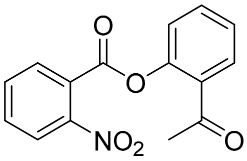	285.26	2´-Hydroxy-acetophenone-ester

MPK701	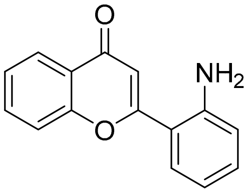	237.26	Flavone
MPK733	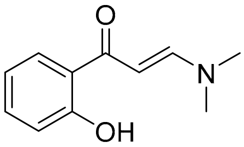	191.23	Chalcone
MPK741	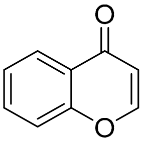	146.15	Flavone
MPK611	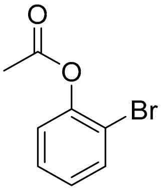	215.05	Phenolester
MPK610	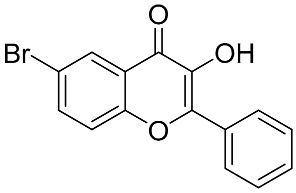	317.14	Flavonol
MPK630	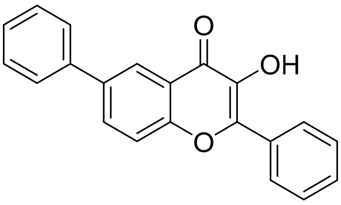	314.34	Flavonol
MPK746	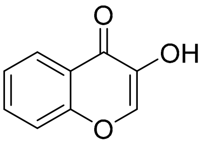<br>Molecular Weight: 162,14	162.14	Flavonol
FFK15	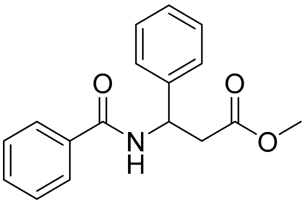<br>Molecular Weight: 283,33	283.33	β-amino acid ester

FFK17	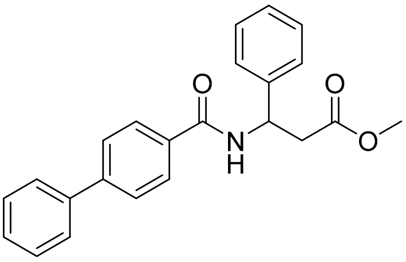<br>Molecular Weight: 359,43	359.43	β-amino acid ester
FFK14	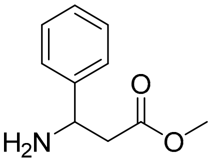<br>Molecular Weight: 179,22	179.22	β-amino acid ester
FFK21	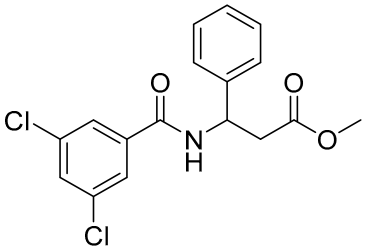<br>Molecular Weight: 352,21	352.21	β-amino acid ester
FFK20	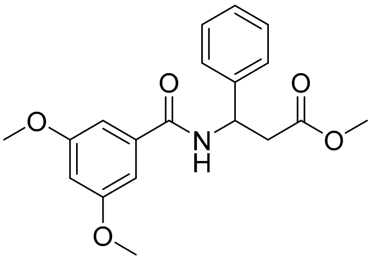<br>Molecular Weight: 343,38	343.38	β-amino acid ester
YSKK53	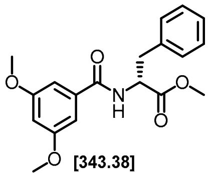 [343.38]	343.38	α-amino acid ester
YSKK54	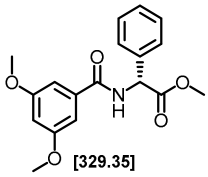 [329.35]	329.35	α-amino acid ester
YSKK55	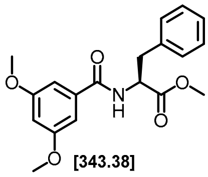 [343.38]	343.38	α-amino acid ester

YSKK 61	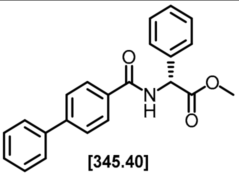 [345.40]	345.40	α-amino acid ester
YSKK60	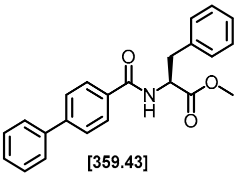 [359.43]	359.43	α-amino acid ester
YAK318	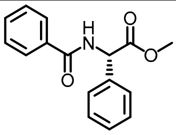 [269.30]	269.30	α-amino acid ester
YAK494	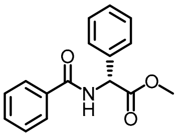 Molecular Weight: 269,30	269.30	α-amino acid ester
YAK322	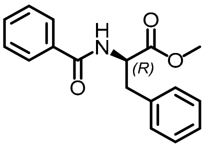 [283.33]	283.33	α-amino acid ester
YAK493	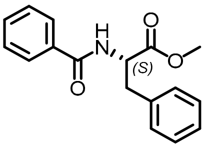 [283.33]	283.33	α-amino acid ester
YAK498	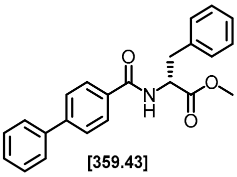 [359.43]	359.43	α-amino acid ester
BLK139	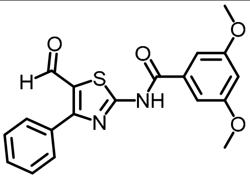 Molecular Weight: 368,41	368.41	Thiazole derivative

BLK157	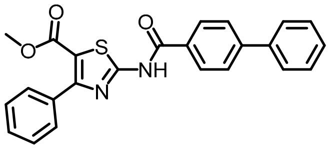<br>Molecular Weight: 414,48	414.48	Thiazole derivative
